# The power of one: A single flanker produces compatibility effects in the episodic flanker task

**DOI:** 10.3758/s13421-024-01653-1

**Published:** 2024-10-29

**Authors:** Gordon D. Logan, Dakota R. B. Lindsey, Jana E. Ulrich

**Affiliations:** 1 Vanderbilt University, Nashville, TN USA; 2 University of South Alabama, Mobile, AL USA

**Keywords:** Flanker task, Selective attention, Memory retrieval, Spotlight model

## Abstract

The episodic flanker task is an episodic version of the Eriksen and Eriksen (*Perception & Psychophysics*, *16* (1), 143–149, 1974) perceptual flanker task, showing the same compatibility and distance effects. Subjects are presented with a list followed by a probe display in which one item is cued. The task, to indicate whether the probed letter appeared in the same position in the memory list, requires focusing attention on a single item in memory. The probe display contains flanking items to be ignored. They are *same* as the memory list or *different*. *Same* flankers are compatible with “yes” responses and incompatible with “no” responses. *Different* flankers are incompatible with “yes” responses and compatible with “no” responses. Previously, we presented multiple flankers in the probe, allowing a global matching strategy. Here, we report two episodic flanker experiments with just one flanker in the probe to encourage focusing sharply on the target. We found flanker compatibility effects in both experiments when a single flanker appeared immediately adjacent to the target. Experiment 2 varied the distance between the flanker and the target in the probe and the memory list and found the compatibility effect in response time only when the flanker was immediately adjacent to the target in both the probe and the memory list. The effect in accuracy also appeared when the flanker was two positions away in both the probe and the memory list. These results show that attention is focused sharply on elements of a memory structure during retrieval, suggesting that memory retrieval is perceptual attention turned inward.

## Introduction

Attention is often described as a spotlight that focuses on desired information and excludes the rest. Things that fall within the spotlight are processed; things that fall outside it are not (Eriksen & Eriksen, 1974). Focusing the spotlight improves signal detection (Posner, 1980) and binds the features of the objects it is focused on (Treisman & Gelade, 1980). Like many before us, we propose that memory retrieval involves turning the same spotlight inward to focus on desired information in memory: memory retrieval is attention turned inward (Broadbent, 1957; Cowan, 1988; Craik & Tulving, 1975; James, 1890; Nobre et al., 2004; Norman, 1968; Rugg et al., 2008). We have evaluated this proposal by implementing tasks that tap different aspects of perceptual attention (attention turned outward) as memory tasks that require selective retrieval (attention turned inward). We fit the data with computational models of memory retrieval, interpreting their retrieval cues as spotlights of attention turned inward (Logan et al., 2021). Empirically, we have shown that memory retrieval produces the same pattern of dual task interference as perceptual attention (Logan et al., 2023a), the same time-course of focusing attention on a specific item in memory as in perception (Logan et al., 2023b), and the same pattern of compatibility and distance effects as perceptual attention in an episodic version of the Eriksen and Eriksen (1974) flanker task (Logan et al., 2021, 2023b). This article extends the parallel between perceptual and episodic flanker tasks, asking whether a single flanker can produce compatibility effects, and providing a new measure of distance that more closely parallels the distance manipulation in the perceptual flanker task.

The perceptual flanker task was designed to measure the sharpness of the focus of attention, on the hypothesis that things that fall within the spotlight are processed and things that fall outside it are not (Eriksen & Eriksen, 1974; Eriksen & Hoffman, 1972). Subjects are shown a central target surrounded by flankers and are asked to classify it (e.g., HHSHH, where the underline represents the target). *Compatible* flankers would require the same response as the target, shortening response time (RT) and increasing accuracy; *incompatible* flankers would require the opposite response, lengthening RT and decreasing accuracy. The distance between the flankers and the target is manipulated to measure how sharply perceptual attention is focused (e.g., HH S HH). Distant flankers fall outside the spotlight and so produce smaller compatibility effects.

The episodic flanker task was designed to test the same hypotheses in memory retrieval (Logan et al., 2021). It requires focusing attention on a target item in memory while ignoring flanking items in the list, and the sharpness of the focus is a central question. Subjects are given a list of letters to remember (ABCDEF) followed by a probe display that contains the same number of letters, one of which is cued by a caret (^) presented underneath it. Their task is to indicate whether the letter in the cued position in the probe occupied the same position in the memory list. Probe letters that require a “no” response are sampled from other positions in the list, so subjects have to focus on the cued position to respond correctly. The flanking letters in the probe are either the *same* as the letters on the memory list (ABCDEF, where the underline represents the caret cue) or *different* from them (STCVXY). Same-context probes are compatible with “yes” responses (ABCDEF) and incompatible with “no” responses (ABDCEF). Different-context probes are incompatible with “yes” responses (STCVXY) and compatible with “no” responses (STDVXY). The flanker effect is *episodic* because compatibility is defined with respect to the list, and the list changes on every trial.

Accurate performance on the episodic flanker task requires a *local match* strategy, in which attention is focused on the position of the cued item, because “no” items are sampled from uncued positions in the memory list (Logan et al., 2021). The local match strategy produces compatibility effects: focusing on the cued position in memory activates the cued item and activates its neighbors in proportion to their distance from the cued position. All the items that fall within the spotlight are processed (Eriksen & Eriksen, 1974), so neighbors from same-context probes will facilitate “yes” responses and impair “no” responses, while neighbors from different-context displays will facilitate “no” responses and impair “yes” responses. In our experiments RT was shorter and accuracy was higher for compatible responses than for incompatible responses, conceptually replicating the Eriksen and Eriksen (1974) perceptual flanker effect and supporting our conjecture that memory retrieval is perceptual attention turned inward (Logan et al., 2021, 2023b).

Our previous experiments with the episodic flanker task used six-letter probes, which included one cued target and five flanking letters. In same-context probe displays, the flankers repeated the study list (ABCDEF, ACBDEF). In different-context probe displays, the flankers were completely new letters (STCVXY, STBVXY). These probes allow a *global match* strategy, in which subjects base their responses partly on the match of the entire probe display to the memory list. The global match strategy would produce compatibility effects: Same-context displays would match the memory list and provide evidence for a “yes” response, reducing RT and increasing accuracy for “yes” probes and increasing RT and reducing accuracy for “no” probes. Different-context displays would not match the memory list, and so provide evidence for a “no” response that would reduce RT and increase accuracy for “no” probes and increase RT and reduce accuracy for “yes” probes. Logan et al. (2021) distinguished global and local matches by fitting computational models to the data to estimate the weight placed on global and local matches in the decision process. The weight on local matches was much larger, but the weight on global matches was significant.

Our interest in parallels between attention and memory retrieval leads us to think of global and local matches as strategies subjects can implement voluntarily, as different ways of attending to memory. Certainly, subjects can comply with instructions to make either global (does the whole array match?) or local (does the cued letter match?) judgments in memory tasks. In our experiments, global matches would produce errors on incompatible trials but they may speed up performance on compatible trials enough to warrant voluntary reliance on the strategy some of the time (Logan et al., 2021). Alternatively, global matching could be obligatory, like reading the word in the Stroop task. Our research does not distinguish these possibilities.

We report two experiments on episodic flanker tasks with one flanker instead of five in the probe displays. Reducing the number of flankers should reduce the appeal of the global match strategy and encourage more complete reliance on the local match strategy. It allows us to ask new questions that reveal new properties of the spotlight of attention focused on memory: Experiment 1 asks whether a single flanker can produce compatibility effects. Experiment 2 replicates Experiment 1 and varies distance between the flanker and the target to ask how sharply attention is focused on memory.

## Experiment 1: Single flanker

The first experiment was designed to determine whether a single flanker is sufficient to produce the episodic flanker compatibility effect. Subjects were given lists of six random letters to remember followed by probes that contained four hash marks and two letters, one of which was the cued target and one of which was the flanker (e.g., list: ABCDEF, probe ##CD##, where the underline represents the caret ^ we used to cue the target’s position). Their task was to decide quickly and accurately whether the cued letter appeared in the same position in the memory list. There were two types of flankers, same and different. *Same* flankers were sampled from the same memory list as the target. They should provide evidence for a “yes” response, which should reduce RT and increase accuracy for “yes” probes and increase RT and reduce accuracy for “no” probes. *Different* flankers were sampled from the letters that were not in the memory list. They should provide evidence for a “no” response, increasing RT and reducing accuracy for “yes” probes while decreasing RT and increasing accuracy for “no” probes. The compatibility effect is the combination of these predictions: a crossover interaction between same versus different flankers and “yes” versus “no” responses.

In theory, perceptual (Eriksen & Eriksen, 1974) and episodic (Logan et al., 2021) compatibility effects occur because the flankers fall within the spotlight of attention focused on the target. To ensure that the flanker in the probe would fall within the spotlight, we placed it immediately to the left or right of the target. To ensure that the flankers in the memory list would fall within the spotlight on the cued position, *same* flankers were sampled from positions immediately to the left or right of the cued position in the memory list. *Different* flankers were not in the memory list.

In theory, the location of the flanker relative to the target may affect the speed and accuracy of orienting attention to the location of the target in the probe and in memory, it may affect the information that is sampled from the cued location after attention is oriented, or it may affect both. Subjects may orient attention by scanning through the list from left to right (Logan et al., 2021, 2023a). If so, they would encounter a flanker on the left before the target, and it may take them some time to reject it and move on to the target. That would increase RT and possibly decrease accuracy relative to a flanker on the right, which would not be encountered in the left-to-right search for the target. Flanker location could also affect RT and accuracy after attention is focused on the target location. Left-to-right access would activate flankers to the left of the target, which would prime a “yes” response and enhance the compatibility effect relative to flankers to the right of the target. These effects are distinguishable: Orienting time and decision time are separable (Logan et al., 2023b). In orienting, flanker location should affect RT and accuracy without changing the compatibility effect. In deciding, flanker location should change the compatibility effect without affecting RT and accuracy overall.

### Method

#### Subjects

We tested 32 subjects from the subject pool at Vanderbilt University. All subjects completed the consent process before beginning the experiment. The procedures were approved by the Vanderbilt University Institutional Review Board.

#### Apparatus and stimuli

The experiment was run in E-Prime 2.0 (Psychology Software Tools, 2012) on ASUS M32BF desktop computers with BenQ XL2411Z flat-screen monitors. Each subject was tested individually in a private testing room in the laboratory. The letters in the memory and probe displays were the 20 consonants, excluding Y, rendered in capitals in Courier New font, size 26 pt. Responses were taken from the Z and M keys on standard QWERTY keyboards, which were the only responses the program accepted.

**Fig. 1:**
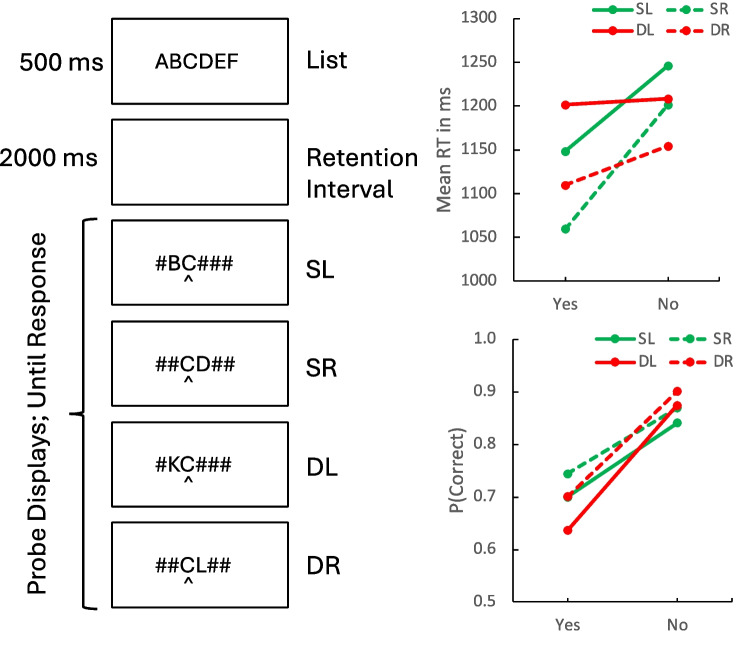
The events on a trial (**left**) and the results (**right**) of Experiment 1. The events panel shows examples of the four types of probes: Same context, flanker on left (SL); same context, flanker on right (SR); different context, flanker on left (DL); and different context, flanker on right (DR). The results panel shows mean response time (RT) (**top**) and proportion correct (**bottom**) for the four types of probes as a function of response type (“yes” vs. “no”). Flankers on the left (SL, DL) are plotted with solid lines. Flankers on the right (SR, DR) are plotted with dashed lines

The events on each trial are shown below in Fig. 1. Each trial began with a fixation cross ( +) centered horizontally and vertically on the screen, which was presented for 500 ms. It was replaced by a six-consonant memory list, which was also centered horizontally and vertically on the screen (e.g., XTMDVP). A different set of six unique memory letters was sampled at random for each trial for each subject. The memory list was displayed for 500 ms and replaced by a blank screen for 2,000 ms. Then the probe display appeared and remained on the screen until the subject responded. The probe display consisted of one target letter cued with a caret (^) cue underneath it, one flanker letter immediately to the left or right of the target, and four # symbols (e.g., ##CD##). *Same* flankers were sampled from the corresponding position in the memory list, immediately left or right of the target position. *Different* flankers were sampled randomly from the 14 consonants not used in the memory list. “No” items were sampled from the memory list two positions to the left or right of the target position. Flankers to the left were not possible at the beginning of the list and flankers to the right were not possible at the end of the list. When this occurred, we “reflected” the flanker, so the flanker that would have appeared to the left when the first position was cued would now appear to the right of the cued position. This affected only the first and the last positions in the probe. We analyzed the data including and excluding the end positions and found the same results in the means and the inferential statistics, so we decided to report the analysis that included all positions.

The probe display was centered horizontally and vertically, and the monospace font made spacing identical to the memory display. When the subject responded, the screen went blank for 500 ms, after which the fixation point for the next trial appeared.

#### Procedure

The basic design of the experiment involved 48 trials, in which each serial position was probed eight times, once for each combination of two flanker positions (left, right), two context types (same, different), and two response types (“yes,” “no”). There were ten replications of the basic design for a total of 480 trials divided into five blocks of 96. Each block consisted of two replications of the basic design presented in random order. The experiment began with instructions and a set of 12 practice trials that were identical in structure to the experimental trials and probed each serial position twice. All subjects saw the same practice trials but the order was randomized separately for each subject.

The instructions described the sequence of displays and the task, including the stimulus to response mapping, which was counterbalanced across subjects with half pressing Z for “yes” and M for “no” and half doing the opposite. Subjects were encouraged to respond as quickly and accurately as possible. Breaks were encouraged between blocks of 96 trials.

#### Data analysis

The 2 (response type: “yes” or “no”) × 2 (context: *same* or *different*) × 2 (flanker location: left or right) design of the experiment fit nicely into a factorial analysis of variance (ANOVA). The main focus of the analysis was the compatibility effect, which is the interaction between response and context and the higher order interactions it participates in. We hypothesized a specific crossover pattern in the interaction, in which RTs were shorter and accuracy was higher for same-context “yes” probes and different-context “no” probes than for different-context “yes” probes and same-context “no” probes. We tested our hypothesis with a planned contrast using weights {-1 1 1 -1} for same-context “yes,” same-context “no,” different-context “yes,” and same-context “no” trials, respectively, multiplying the means in each condition by the contrast weight and summing the products over conditions. A positive contrast value indicates the predicted crossover interaction. The standard error of the contrast can be expressed numerically, but it cannot be expressed meaningfully as error bars around the points that comprise the interaction in a graph. It expresses variability in the pattern of the points, not variability in specific points. Error bars around specific points would not support statistical inferences about the crossover pattern. Consequently, we did not put error bars in our figures.

Subject 13 had very low accuracy in all conditions. It appeared that they had reversed the instructed mapping of “yes” and “no” responses onto the response keys. When we scored their data with the mapping reversed, their accuracy was acceptable, so we included their data scored this way in the analyses. We also performed the analyses excluding Subject 13’s data. The results were very similar and led to the same conclusions. We report data and analyses from all 32 subjects, including Subject 13.

### Results and discussion

The mean RTs and proportions of correct responses for *same-* and *different-*context trials are plotted as a function of response (“yes” vs. “no”) in Fig. 1. Contrasts evaluating the compatibility effect are presented in Table 1. Both RT and accuracy show the crossover interaction between context and response type that defines the compatibility effect. This is the main result of the experiment: The episodic flanker compatibility effect can be replicated with a single flanker. The effect was robust. The contrast was positive in 27/32 subjects for both RT and accuracy. It was significant for RT [*t*(31) = 4.8860, *p* = 0.00003, *SEM* = 38.6296] and accuracy [*t*(31) = 5.1598, *p* = 0.00001, *SEM* = 0.0333].

The compatibility effects were similar in size to the compatibility effects observed in previous experiments with five flankers. Table 1 contains the mean compatibility contrasts for RT and accuracy from Experiments 2–5 of Logan et al. (2021) and Experiments 2a and 2b of Logan et al. (2023b), which probed memory with five flankers. These contrasts were calculated for “yes” items (lag 0) and “no” items with lag 2, as in the present experiment. For RT, the compatibility effects with one flanker were about the same as the effects with five flankers in Logan et al. (2021) and somewhat larger than the effects in Logan et al., (2023a, b). For accuracy, the compatibility effects in the present experiments were smaller than the effects in Logan et al. (2021) and about the same as the effects in Logan et al., (2023a, b). Overall, the results suggest that subjects may not have relied much on the global match strategy in Logan et al. (2021) and Logan et al., (2023a, b). The compatibility effects in those studies are not much different from those in the present experiments, which precluded the global matching strategy by presenting single flankers.

**Table 1:** Mean compatibility contrast values for response time (RT) and accuracy in Experiment 1 and the M1P1 condition of Experiment 2, which presented one flanker, and in Experiments 2–5 from Logan et al. (2021) and Experiments 2a and 2b from Logan et al. (2023b), which presented five flankers

Source	Experiment	RT	SEM	P(Correct)	SEM
Present experiments	1	189	39	.1719	.0333
	2 M1P1	214	37	.1892	.0369
Logan et al. (2021)	2	208	37	.2944	.0293
	3	197	30	.2897	.0294
	4	151	37	.2569	.0342
	5	228	51	.2867	.0397
Logan et al. (2023b)	2a	128	13	.2022	.0281
	2b	124	18	.2165	.0356

SEM = standard error of the mean. Compatibility contrast for RT = -1 × Same Yes + 1 × Same No + 1 × Different Yes – 1 * Different No. The signs are reversed for the compatibility contrast for P(Correct). Contrasts for Logan et al. (2021) and Logan et al. (2023b) were calculated comparing “yes” responses with lag 2 “no” responses, comparable to the design in the present Experiment 1

The location of the flanker had strong effects on RT and accuracy. Responses with flankers on the left were 63 ms longer and 0.0369 less accurate than responses with flankers on the right. However, the compatibility effect on RT and accuracy was about the same whether the flanker was on the left or the right. This suggests that flanker location affected orienting to the cued location but not deciding about the match after attention was oriented (Logan et al., 2023b). “Yes” responses were faster and less accurate than “no” responses, which is typical of episodic flanker experiments (Logan et al., 2021, 2023b). The models in Logan et al. (2021) accounted for this with a lower best-fitting response threshold in the decision process for “yes” responses than for “no” responses.

These conclusions were supported by 2 (response type: “yes” or “no”) × 2 (context: *same* or *different*) × 2 (flanker location: left or right) ANOVAs on the mean RTs and proportions of correct responses for each subject. The summary tables are displayed in Table 2. The main effects of response type and flanker location were significant in both measures. The interactions between response type and context, assessing the compatibility effect, were significant in both measures. The three-way interaction between response type, context, and flanker location was not significant in either analysis, confirming the conclusion that the compatibility effect was not altered by flanker location.

**Table 2:** Summary tables for 2 (response “yes” or “no”) × 2 (context same or different) × 2 (flanker on the right or left) analyses of variance on mean response time (RT) and proportion of correct responses in Experiment 1

	*F*(1,30)	*MSError*	*p*	${\eta }_{p}^{2}$
RT				
Response type (R)	12.6524	17,683.1152	.0013	.2966
Context (C)	.0170	8604.4993	.8971	.0006
Flanker (F)	43.4835	5876.7089	< .0001	.5861
R × C	22.1661	6164.1025	< .0001	.4249
R × F	5.0822	5360.5209	.0316	.1449
C × F	.1507	3669.9117	.7006	.0050
R × C × F	.3159	5188.0337	.5783	.0104
P(Correct)				
Response type (R)	44.8130	.0460	< .0001	.5990
Context (C)	3.4684	.0023	.0724	.1036
Flanker (F)	10.4989	.0080	.0029	.2592
R × C	24.0339	.0045	< .0001	.4448
R × F	1.8452	.0044	.1845	.0579
C × F	.3719	.0025	.5466	.0122
R × C × F	1.2525	.0018	.2720	.0401

## Experiment 2: Flanker distance

The second experiment was a replication of the first with a new manipulation of flanker distance to test the sharpness of the focus of attention on memory. Our previous experiments tested the sharpness of the focus by presenting “no” items from other positions in the memory list and varying the distance in the list between the cued position and the “no” item. The shorter the distance, the more the “no” item would fall within the spotlight and prime a “yes” response, increasing RT and decreasing accuracy (Logan et al., 2021). Experiment 2 tested the sharpness of the focus by varying the distance between the flanker and the cued item in the probe, like the distance manipulation in the Eriksen and Eriksen (1974) flanker task. The flanker was placed ± 1 or ± 2 positions away from the cued location (probe lag). *Same* flankers were sampled from ± 1 or ± 2 positions away from the cued location in the memory list (memory lag). Memory lag was undefined for *different* flankers.

Flankers in positions adjacent to the target in the probe and the memory list (probe lag ± 1, memory lag ± 1) replicate the conditions of Experiment 1. The other combinations of probe lag and memory lag test the sharpness of the focus of attention on the cued positions. Probe and memory lags of ± 2 may fall farther outside the spotlight and produce smaller compatibility effects. Such results would suggest that the single flankers disable the global match strategy that was possible in the previous experiments with five flankers, so that performance depends only on the local match at the cued position (Logan et al., 2021).

### Method

#### Subjects

We tested 32 subjects from the subject pool at Vanderbilt University. All subjects completed the consent process before beginning the experiment. The procedures were approved by the Vanderbilt University Institutional Review Board.

#### Apparatus and stimuli

**Fig. 2:**
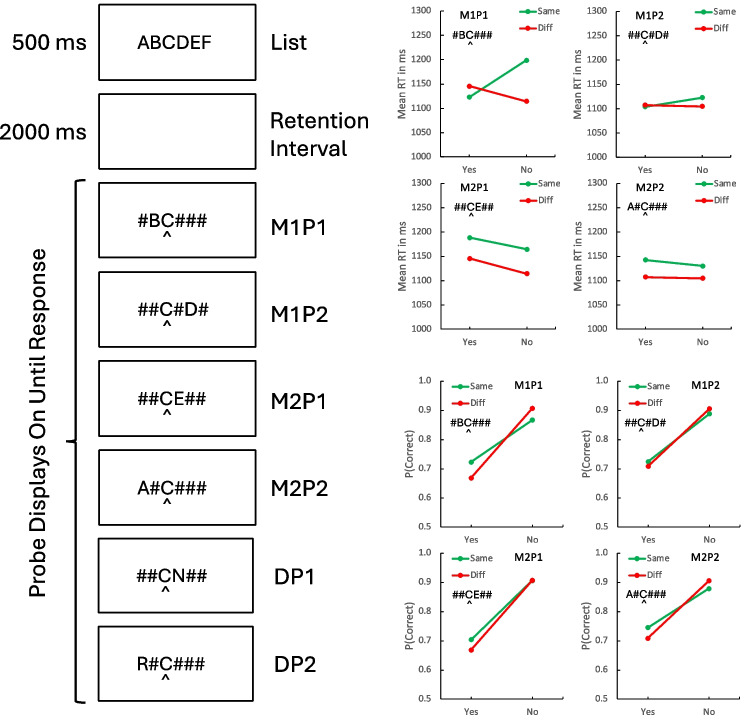
The events on a trial (**left**) and the results (**right**) of Experiment 2. The events panel shows examples of the six types of probes. The four same context probe types are the factorial combination of memory lag (M1, M2) and probe lag (P1, P2). The two different context probe types vary probe distance (DP1, DP2). The results panel shows the compatibility contrast between context (Same, Diff) and response type (Yes, No) for each combination of memory lag and probe lag. An example of the relevant probe type for Same trials is inset in each panel

These were the same as in Experiment 1, except for the sampling of flanker locations in the probes and in the memory lists. Subjects were tested individually in the laboratory. “No” items were sampled from the memory items that remained after the cued item and the flanker were selected. Given list ABCDEF and C and D as the cued item and flanker, respectively, a “no” item would be sampled randomly from A, B, E, and F. The events on each trial appear below in Fig. 2. As in the previous experiment, not all memory and probe directions and lags were available at all positions. Left was undefined for serial position 1 and right was undefined for serial position 6, so we reflected them, as before. Negative memory and probe lags were undefined at the beginning of the list and positive lags were undefined at the end of the list. They, too, were reflected. This affected positions 1, 2, 5, and 6, so we analyzed the data including and excluding those positions. Again, we found the same patterns in the means and in the inferential statistics, so we report the analyses including all positions in this article.

#### Procedure

The basic design required 192 trials. “Yes” trials required 2 (context same or different) × 2 (flanker left or right) × 2 (memory lag 1 or 2) × 2 (flanker lag 1 or 2) × 6 (serial positions) = 96 trials. “No” trials required 2 (context same or different) × 2 (flanker left or right) × 2 (probe lag 1 or 2) × 6 (serial positions) = 48 trials. The 48 “no” trials were replicated to equate the number of “yes” and “no” trials in the basic design. There were three replications of the basic design for a total of 576 trials. Breaks were given every 96 trials. The instructions were the same as in Experiment 1.

#### Data analysis

The design of the experiment did not fit nicely into a factorial ANOVA. The memory distance factor was undefined for *different* responses. Consequently, we analyzed the data with planned contrasts. One set of contrasts examined the compatibility effect (response × context interaction) in each combination of memory lag and probe lag. The same *different* context data were used both in the contrasts evaluating memory lag 1 and the contrasts evaluating memory lag 2. Another set of contrasts evaluated the effects of memory lag, probe lag, response, and flanker location. We also calculated contrasts in the memory lag 1, probe lag 1 condition to compare compatibility effects for flankers on the left and flankers on the right. The most important results are the compatibility contrasts evaluating the interaction between response and context. The standard errors of the contrasts cannot be expressed meaningfully as error bars around the points that comprise the interaction in a graph, so we did not put error bars in our figures.

### Results and discussion

The mean RTs and proportions of correct responses for *same-* and *different-*context trials are plotted as a function of response (“yes” vs. “no”) in Fig. 2. Different panels represent different combinations of memory lag and probe lag. Memory lag was undefined for *different* probes, so the *different* data in the plots for memory lag 1 and memory lag 2 are the same.

The results for memory lag 1 and probe lag 1 replicate the compatibility effect observed in Experiment 1: “Yes” RTs were shorter and accuracy was higher when the context was the *same* as the memory list than when it was *different*. “No” RTs and accuracy showed the opposite effect, producing the signature crossover interaction. The compatibility effect was weaker for the other combinations of memory lag and probe lag, suggesting a sharp focus on the cued position and the items immediately surrounding it that excludes more distant items. We assessed these effects with planned contrasts in each combination of memory lag and probe lag. The results are presented in Table 3. For RT, the compatibility contrast was significant only for memory lag 1 and probe lag 1. It was not significant for any other combination. For proportion correct, the compatibility contrast was significant for memory lag 1 and probe lag 1 and for memory lag 2 and probe lag 2 but not for memory lag 1 and probe lag 2 and for memory lag 2 and probe lag 1. This is a hint of the global match strategy: Accuracy was higher when memory lag and probe lag matched than when they differed.

**Table 3:** Contrasts evaluating compatibility effects in response time (RT) and proportion correct in each combination of memory lag (M1, M2) and probe lag (P1, P2) in Experiment 2

	*t*(31)	*SEM*	*p*	*Cohen’s d*
RT				
M1P1	5.7640	37.0580	< .0001	1.0189
M1P2	.2412	59.8377	.8110	.0426
M2P1	.8277	51.4700	.4142	.1463
M2P2	.3991	48.81	.6925	.0706
P(Correct)				
M1P1	5.1236	0.0369	< .0001	.9057
M1P2	1.6216	0.0444	.1150	.2867
M2P1	1.3906	0.0472	.1743	.2458
M2P2	2.4624	.0512	.0196	.4353

SEM = standard error of the mean. Compatibility contrast for RT = -1 × Same Yes + 1 × Same No + 1 × Different Yes – 1 * Different No. The signs are reversed for the compatibility contrast for P(Correct)

RT was affected more by the distance between the flanker and the target in the probe display than by the distance in the memory list. RT was 44 ms longer for probe lag 1 than for probe lag 2 and 19 ms shorter for memory lag 1 than for memory lag 2. Contrasts presented in Table 4 show that the distance effect was significant for probe displays but not for memory lists. A contrast evaluating the interaction between probe distance and memory distance was not significant. Accuracy was not affected by distance in the probe or distance in the memory list. “Yes” responses were no faster than “no” responses, but they were less accurate. RT was 77 ms slower for flankers on the left than for flankers on the right and the difference was significant (Table 5). Accuracy was 0.0258 lower for flankers on the left but the difference was not significant (Table 5).

**Table 4:** Contrasts evaluating effects of memory lag (M = 1 or 2), probe lag (P = 1 or 2), response type (“yes” or “no”), and flanker location (left or right) in Experiment 2

	*t*(31)	*SEM*	*p*	*Cohen’s d*
RT				
Memory lag	1.8204	84.2601	.0784	.3218
Probe lag	4.1717	83.8273	.0002	.7375
M × P	.4320	67.3391	.6687	.0764
Response	.7724	150.5981	.4457	.1365
Flanker location	3.6637	185.9619	.0009	.6476
P(Correct)				
Memory lag	.9287	.0660	.3602	.1642
Probe lag	1.3175	.0560	.1973	.2329
M × P	.2484	.0622	.8055	.0439
Response	7.9866	.1609	< .0001	1.4119
Flanker location	1.8304	.1033	.0768	.3236

RT = response time, SEM = standard error of the mean

**Table 5:** Contrasts evaluating compatibility and flanker location effects in response time (RT) and proportion correct in Experiment 2 for trials in which memory lag and probe lag are both 1 (replication of Experiment 1). Comp Right tests the compatibility effect for flankers to the right of the target. Comp Left tests the compatibility effect for flankers to the left of the target. Comp R-L tests the difference in compatibility effects for flankers to the left and right. Flanker location compares overall RT and proportion correct for flankers on the right and left

	*t*(31)	*SEM*	*p*	*Cohen’s d*
RT				
Comp right	4.4781	23.1259	.0001	.7916
Comp left	3.6892	29.8288	.0009	.6522
Comp R-L	.1688	38.4165	.8671	.0298
Flanker location	5.2232	45.2730	< .0001	.9233
P(Correct)				
Comp right	3.4887	.0256	.0015	.6167
Comp left	3.8326	.0260	.0006	.6775
Comp R-L	.7783	.0361	.7783	.0502
Flanker location	1.2110	.0330	.2351	.2141

SEM = standard error of the mean

**Fig. 3:**
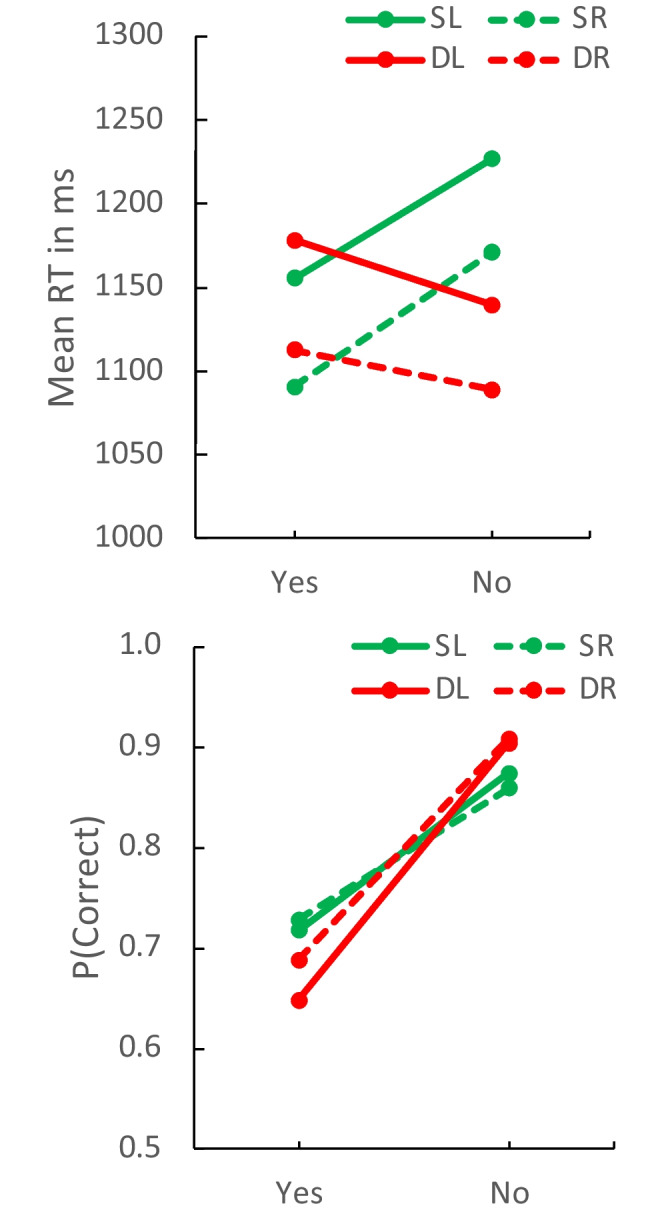
Mean response time (RT) (**top**) and proportion correct (**bottom**) for same (S) and different (D) contexts as a function of response type (Yes, No) for flankers presented on the left (SL, DL; solid lines) and flankers presented on the right (SR, DR; dashed lines) in Experiment 2

The memory lag 1 and probe lag 1 data replicate the effects of flanker position (left and right) and their null interaction with compatibility in Experiment 1. The mean RTs and proportions of correct responses, plotted in Fig. 3, show the same crossover interactions for left and right flanker trials as Fig. 1, with left trials shifted up for RT and down for accuracy. The mean compatibility contrasts for RT and accuracy for memory lag 1 and probe lag 1, shown in Table 1, were close to the values from Experiment 1 and about as large the mean values from Logan et al. (2021) and Logan et al. (2023b). The null compatibility effects for more remote flankers suggest that the focus of attention in a local match includes the target and its immediate neighbors and excludes the rest. Thus, the compatibility effects with five flankers may depend mostly on the local matching strategy.

Table 5 contains contrasts showing that the compatibility contrasts were significant for flankers on the left and the right for both RT and accuracy and that the differences between the left and right contrasts were not significant. Table 5 also shows significantly longer RTs for flankers on the left than for flankers on the right, as in Experiment 1. Accuracy was lower for flankers on the left but the difference was not significant.

## General discussion

The experiments showed that a single flanker placed next to the target in the episodic flanker task produces compatibility effects analogous to the flanker compatibility effects in the Eriksen and Eriksen (1974) perceptual flanker task. This result strengthens the analogy between episodic and perceptual flanker tasks and supports the conjecture that memory retrieval is selective attention turned inward (Logan et al., 2021). Experiment 2 showed that the compatibility effect in RT occurred only if the flanker was adjacent to the target in both the probe and the memory list. It occurred in accuracy when the flanker occupied the same position in the probe and the memory list (both ± 1 or both ± 2 from the probe position). The RT results indicate a sharp focus on the cued item: only the target and its immediate neighbors fall within the spotlight. The accuracy results suggest a role for a global match.

These compatibility effects converge nicely with the results from our previous experiments with six-item lists and five-flanker probes (Logan et al., 2021, 2023b). The effects were about the same with one flanker as with five (Table 1). This suggests that the compatibility effects with five flankers reflect the use of the local match strategy more than the global match strategy, consistent with conclusions Logan et al. (2021) drew from modeling the two strategies. The weight of the local match was much greater than the weight on the global match in the decision process.

Experiment 2 employed a novel distance manipulation, presenting the flanker one or two positions to the left or right of the target in the probe. Our previous experiments manipulated distance by varying the “no” letters in the cued position in the probe. Given list ABCDEF, the probe STBXVR is distance -1 and the probe STEXVR is distance 2 (Logan et al., 2021, 2023b). RT decreased and accuracy increased with the distance of the “no” probe, directly analogous to the effects of distance between the flanker and the target in the probe in the present experiments. Both results suggest a sharp focus on the cued position in the probe, as attention is turned outward, and a sharp focus on the cued position in memory, as attention is turned inward.

Both experiments showed longer RT and lower accuracy when the single flanker appeared on the left of the target than when it appeared on the right, but the compatibility effects were about the same regardless of flanker location. This suggests that flanker location affected the time to orient attention to the target position in memory but did not affect the decision process once attention was focused on the target position. Logan et al. (2023b) found similar null interactions between the compatibility effect and the delay of a pre-cue indicating the target’s position, suggesting that pre-cue delay affected orienting but not deciding after attention was focused on the target. As in perceptual attention (Posner, 1980), the distinction between orienting and deciding is important in attention to memory. We know a lot about the decision process, especially if we extend our scope to include the decision processes in memory models (e.g., Logan et al., 2021). We know a lot less about the orienting process in memory retrieval. Rectifying that imbalance is an important direction for future research.

We had no a priori hypotheses about the difference between flankers on the left and flankers on the right. Previous experiments with cued recall from serial lists found shorter RT and higher accuracy when the cues were items to the left of the target in the serial list than when the cues were items to the right (Kahana & Caplan, 2002), so we might expect facilitation, but we found the opposite effect. One possibility is that our result reflects the time to find and encode the target in the probe. If subjects found the probe by scanning the display from left to right (Davis, 2010; Mewhort et al., 1969; Whitney, 2001), they would encounter a flanker on the left and would have to reject it before they reach the target. If the flanker was on the right, they would encounter the target first and not have to reject the flanker. This would account for the RT difference and could account for the accuracy difference if they sometimes failed to reject the flanker and used it to probe memory instead of the target.

**Fig. 4:**
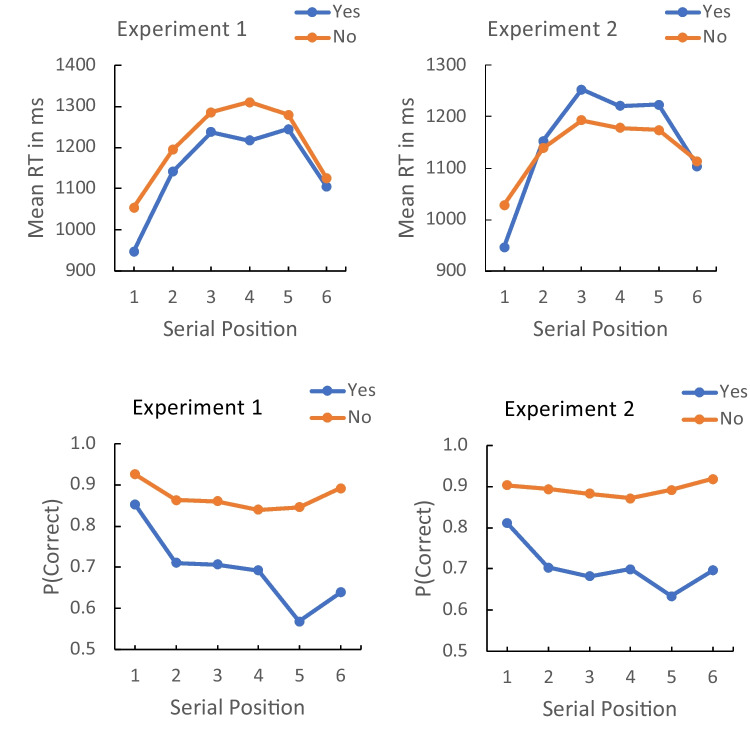
Mean response time (RT) (**top**) and proportion of correct responses (**bottom**) for Yes and No responses as a function of serial position in Experiments 1 (left) and 2 (right)

These speculations led us to examine serial position effects for evidence of the assumed left-to-right scanning. We calculated mean RT and accuracy for “yes” and “no” trials in each experiment, collapsing across context and flanker location to increase the number of observations. The results, presented in Fig. 4, show some evidence of left-to-right scanning in both experiments. The RTs for “yes” and “no” responses increase from positions 1–3, level off for positions 4 and 5, and decrease for position 6 at the end of the list. The accuracies for “yes” responses decrease from positions 1–5 and show a small recency effect at position 6. The accuracies for “no” responses did not change much with position. Contrasts testing the linear and quadratic trends for RT and accuracy for “yes” and “no” responses in each experiment are presented in Table 6. The linear trends were significant for “yes” and “no” responses for RT and for “yes” responses for accuracy in both experiments, consistent with left-to-right scanning. The quadratic trends were significant for both responses in RT and accuracy in both experiments, consistent with a tendency to scan the list from either end to the middle (Fischer-Baum & McCloskey, 2015; Logan et al., 2023a). Altogether, the evidence for left-to-right scanning is at best suggestive. We cannot rule out possible alternatives, like right-to-left (backward) scanning, entirely. Solid conclusions require further research.

**Table 6:** Contrasts assessing linear and quadratic trends in serial position effects in response time (RT) and accuracy data in Experiments 1 and 2

	*t*(31)	*SEM*	*p*	Cohen’s d
Experiment 1 linear trends				
RT Yes	3.3817	321.8632	.0020	.5978
RT No	3.7941	168.7864	.0006	.6707
P(C) Yes	3.4892	.4379	.0025	.6168
P(C) No	1.8345	.1354	.0762	.3243
Experiment 1 quadratic trends				
RT Yes	9.5958	205.9754	< .0001	1.6963
RT No	8.9937	221.0829	< .0001	1.5899
P(C) Yes	2.7168	.2156	.0107	.4803
P(C) No	4.4210	.1332	.0001	.7815
Experiment 2 linear trends				
RT Yes	2.5540	370.6426	.0160	.4587
RT No	2.8934	175.7042	.0070	.5197
P(C) Yes	2.2761	.3305	.0301	.4088
P(C) No	0.8253	.0668	.0668	.1482
Experiment 2 quadratic trends				
RT Yes	.82548	240.5577	< .0001	1.4826
RT No	6.3511	168.5564	< .0001	1.1407
P(C) Yes	3.4336	.1924	.0018	.6167
P(C) No	3.1721	.0935	.0035	.5697

SEM = standard error of the mean

More broadly, our results strengthen the parallels between attention turned outward in the perceptual flanker task and attention turned inward in the episodic flanker task. They extend the conditions under which the critical compatibility and distance effects have been observed and offer a new manipulation of distance that converges with our previous distance manipulation with “no” items. They strengthen the claim that the same computational mechanism is engaged in attention to memory and attention to perception. The mechanisms are clearly analogous. We believe they may be one and the same. That possibility offers an economy of mechanism and the chance to integrate theories of attention and memory computationally and mathematically so one theory explains the two domains simultaneously.

**Fig. 5:**
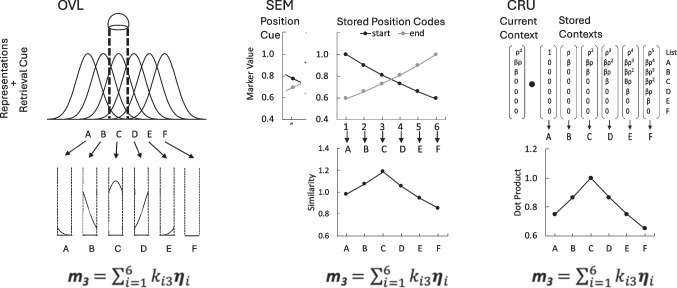
Models of serial order applied to cued recognition when position 3 is cued. OVL (left column) is the overlap model. Items are represented as distributions in space and a “spotlight” selects a region, activating the letters in memory in proportion to the area of their distribution that falls within the spotlight. This results in a retrieved memory vector that includes all the activated items in proportion to their activation. SEM (middle column) is the start–end model. Items are associated with position codes representing the distance from the beginning and the end of the list (top). Position codes cue retrieval, activating other position codes in proportion to their similarity, which activate the items associated with them (middle), resulting in a memory vector with the same form as that of the OVL. CRU (right column) is the context retrieval and updating model. Items are associated with stored contexts that are built from items encountered during encoding. The current context is used to cue retrieval, activating the other contexts in proportion to their similarity, which results in a retrieved memory vector with the same form as that of the OVL and SEM. Decisions are made by comparing the retrieved memory vector with a vector computed from the probe in the same manner. Flanking letters from adjacent positions influence the decision in all three models

As a step in this direction, Logan et al. (2021) applied three models of serial memory to the episodic flanker task, using their retrieval cues and decision process to focus attention on the cued position and decide whether the memory item matched the probe. The three models are depicted in Fig. 5. OVL is the *overlap model* developed to explain serial recall by Lee and Estes (1981) and extended to same-different judgments by Ratcliff (1981), spatial attention by Logan (1996), and letter order in reading by Gomez et al. (2008). Items are represented as distributions in space and the retrieval cue is a “spotlight” selects a region in space (top). Letters *j* are activated in proportion to the area of their distribution *k*_*ij*_ that falls within the spotlight focused on the third position *i* (middle). This results in a retrieved memory vector (***m***_3_) that is the sum of vectors representing the items (***η***_*i*_) multiplied by the proportions (*k*_*ij*_, bottom).

SEM is the *start–end model* developed by Henson (1998) to explain serial recall and extended to free recall by Farrell (2012) and to reading and spelling by Houghton (2018). Items are associated with position codes representing the distance from the beginning and the end of the list (top). The spotlight of attention (retrieval cue) is the code for the third position. It activates the other position codes in proportion to their similarity (distance), which activate the items associated with them (middle). This results in a retrieved memory vector (***m***_3_) that is the sum item vectors(***η***_*i*_) multiplied by the similarities (*k*_*ij*_, bottom).

CRU is the *context retrieval and updating model*. It is an application of the context maintenance and retrieval model of Howard and Kahana (2002) and Polyn et al. (2009), extended to typewriting and serial recall by Logan (2018, 2021, respectively). Items are associated with stored contexts that are built from items encountered during encoding. The spotlight of attention (retrieval cue) is the context representing the third position. It activates the other contexts in proportion to their similarity (dot product), which varies with distance (middle). This results in a retrieved memory vector (***m***_*3*_) that is the sum item vectors(***η***_*i*_) multiplied by the dot products (*k*_*ij*_, bottom).

In all models, the decision is made by calculating the dot product between the retrieved memory vector (***m***_*3*_) and a vector (***p***_*3*_) that is constructed from the cued position in the probe display in the same manner. Flanking letters from positions 2 and 4 (and beyond) produce the compatibility effect, influencing the decision by providing evidence for a “yes” response if they match the memory list and evidence for a “no” response if they mismatch.

The memory models map nicely onto classical approaches to attention, adding depth to the claim that memory retrieval is attention turned inward. OVL selects regions of space like space-based attention models (Eriksen & Eriksen, 1974; Logan, 1996; Posner, 1980; Treisman & Gelade, 1980); SEM selects items in positions like object-based attention models (Duncan, 1984; Kahneman et al., 1992); and CRU selects items selects items by probing memory with a cue like template-based attention models (Bundesen, 1990; Logan, 2002).

Logan et al. (2021) found that OVL, SEM, and CRU fit the data well. That is not good news from the usual perspective of competitive model testing, where the goal is to find the best-fitting model and discard the rest. It is good news from the perspective of integrating research on memory and attention and the idea that memory retrieval is attention turned inward. The retrieval cues from all models act like spotlights of attention focused on memory, producing effects characteristic of attention focused on perception.

## Conclusions

The experiments show that a single flanker can produce compatibility and distance effects in memory retrieval analogous to attentional effects in the perceptual flanker task. They show that attention is focused sharply on memory so that only the target and its immediate neighbors are likely to fall within the spotlight. These results add to our knowledge of the episodic flanker task and strengthen the evidence for the proposition that memory retrieval is attention turned inward, that attention and memory retrieval are one and the same computationally.

## Data Availability

The raw data, analyzed data, and materials are posted on the Open Science Framework at https://osf.io/rbwju/.

## Code availability

The code for running the experiment and analyzing the data is posted on the Open Science Framework at https://osf.io/rbwju/.
